# Supporting community overdose response planning in Ontario, Canada: Findings from a situational assessment

**DOI:** 10.1186/s12889-022-13762-0

**Published:** 2022-07-19

**Authors:** Triti Khorasheh, Caroline Bennett AbuAyyash, Maryam Mallakin, Kate Sellen, Kim Corace, Bernadette Pauly, Daniel Buchman, Michael Hamilton, Nick Boyce, Karen Ng, Carol Strike, Sheena Taha, Heather Manson, Pamela Leece

**Affiliations:** 1 Health Promotion, Chronic Disease and Injury Prevention, Public Health Ontario, Toronto, M5G 1V2 Canada; 2 Dalla Lana School of Public Health, University of Toronto, Toronto, M5T 3M7 Canada; 3 Health Design Studio, Ontario College of Arts and Design (OCAD) University, Toronto, M5T 1W1 Canada; 4 Department of Psychiatry, University of Ottawa, Ottawa, K1N 6N5 Canada; 5 University of Ottawa Institute of Mental Health Research, Ottawa, K1Z 7K4 Canada; 6 Canadian Institute for Substance Use Research, University of Victoria, Victoria, V8N 5M8 Canada; 7 School of Nursing, University of Victoria, Victoria, V8P 5C2 Canada; 8 Campbell Family Mental Health Research Institute, Centre for Addiction and Mental Health, Toronto, M5G 2C1 Canada; 9 Joint Centre for Bioethics, University of Toronto, University of Toronto, Toronto, M5T 1P8 Canada; 10 Institute for Safe Medication Practices Canada, North York, M2N 6K8 Canada; 11 Ontario Harm Reduction Network, Toronto, M4X 1K9 Canada; 12 Toronto Academic Pain Medicine Institute (TAPMI), Women’s College Hospital, Toronto, M5S 1B2 Canada; 13 Canadian Centre on Substance Use and Addiction, Ottawa, K1P 5E7 Canada; 14 Department of Family and Community Medicine, University of Toronto, Toronto, M5G 1V7 Canada

**Keywords:** Opioids, Overdose, Situational assessment, Public health, Capacity building

## Abstract

**Background:**

Many communities across North America are coming together to develop comprehensive plans to address and respond to the escalating overdose crisis, largely driven by an increasingly toxic unregulated drug supply. As there is a need to build capacity for successful implementation, the objective of our mixed methods study was to identify the current planning and implementation practices, needs, and priority areas of support for community overdose response plans in Ontario, Canada.

**Methods:**

We used a situational assessment methodology to collect data on current planning and implementation practices, needs, and challenges related to community overdose response plans in Ontario, consisting of three components. Between November 2019 to February 2020, we conducted ten semi-structured key informant interviews, three focus groups with 25 participants, and administered an online survey (*N* = 66). Purposeful sampling was used to identify professionals involved in coordinating, supporting, or partnering on community overdose response plans in jurisdictions with relevant information for Ontario including other Canadian provinces and American states. Key informants included evaluators, representatives involved in centralised supports, as well as coordinators and partners on community overdose response plans. Focus group participants were coordinators or leads of community overdose response plans in Ontario.

**Results:**

Sixty-six professionals participated in the study. The current planning and implementation practices of community overdose response plans varied in Ontario. Our analysis generated four overarching areas for needs and support for the planning and implementation of community overdose response plans: 1) data and information; 2) evidence and practice; 3) implementation/operational factors; and 4) partnership, engagement, and collaboration. Addressing stigma and equity within planning and implementation of community overdose response plans was a cross-cutting theme that included meaningful engagement of people with living and lived expertise and meeting the service needs of different populations and communities.

**Conclusions:**

Through exploring the needs and related supports for community overdose response plans in Ontario, we have identified key priority areas for building local capacity building to address overdose-related harms. Ongoing development and refinement, community partnership, and evaluation of our project will highlight the influence of our supports to advance the capacity, motivation, and opportunities of community overdose response plans.

**Supplementary Information:**

The online version contains supplementary material available at 10.1186/s12889-022-13762-0.

## Background

Canada continues to face an unprecedented public health crisis of fatal and non-fatal overdose, with approximately 21 deaths per day in 2021 largely driven by the increasing contamination and toxicity of the unregulated drug supply [1]. To respond to the complexity of this crisis, the Canadian Drugs and Substances Strategy (CDSS) indicates that no single sector or intervention is adequate, but rather comprehensive and collaborative approaches are required (e.g., prevention, treatment, harm reduction, enforcement) [2]. In the Canadian province of Ontario, overdose response strategies have been implemented that reflect the comprehensive approach of the CDSS. Additionally, public health units received provincial funding to support the development and implementation of local overdose response plans [3]. Local opioid or overdose response plans are an emerging public health model that involves the coordinated efforts of multiple community stakeholders to implement actions to prevent and address overdose and related harms (e.g., naloxone distribution, access to treatment, supervised consumption services). These plans vary based on the different contexts in which they are applied [4–7]. Across North America, local plans refer to initiatives responding to either opioids or overdose. Throughout this paper, we refer to them as overdose response plans to recognise that drug overdose is the main outcome of interest and that multiple substances are often present when an opioid-related death occurs [1].

As communities set out to develop and implement local overdose response plans, there is a need to understand how best to build capacity for successful implementation. The World Health Organization (WHO) defines capacity building as the “development of knowledge, skills, commitment, structures, systems and leadership to enable effective health promotion” [8]. Capacity building has been recognised as a fundamental component of the sustainable development and implementation of evidence-based public health practices [9–11]. Within various areas of public health, the application of capacity building strategies and techniques has demonstrated success in a range of outcomes including knowledge enhancement, skills, and improvements in practices or polices [12, 13]. In the context of overdose prevention and response, several US state/ Canadian provincial agencies have launched centralised structures to build capacity and advance local overdose response efforts by offering funding, training, technical assistance, and other supports [14–22]. Examples of these centralized supports include the Overdose Emergency Response Centre and the Community Crisis Innovation Fund in British Columbia. In the Australian context, the Alcohol and Drug Foundation supports Local Drug Action Teams by providing research, resources, and expertise for Community Action Plans on alcohol and other drugs [23]. Preliminary analyses have shown some positive outcomes for community overdose response plans that have received centralised supports including increases in local implementation of evidence-based strategies [15] and decreases in opioid-related deaths [24]. However, the evidence base for centralised supports for community overdose response plans is limited. There is a need to further understand where gaps exist and what supports are needed for the successful planning and implementation of overdose response plans, particularly within the context of Ontario.

For this reason, we sought to engage key stakeholders to understand the local context and capacity building needs. This step will inform the development and delivery of strategies to build capacity for the planning and implementation of community overdose response plans in Ontario. To become familiar with community overdose response plans, we first conducted a scoping review [25]. Later, we conducted a separate search to identify a complete set of available Ontario plans. Building on this work, we conducted a situational assessment to develop understanding of community overdose response plans. Our guiding research question was: What priority actions should our project pursue to support the work of community overdose response plans in Ontario? The aims of our situational assessment was to understand: (1) current planning and implementation practices and needs; and (2) priority supports for community overdose response plans in Ontario. Subsequently, we hosted a co-design workshop to delve further into the detailed components, prioritisation, implementation, and evaluation of supports which will be reported elsewhere.

## Methods

### Aim

This investigation was undertaken within the context of a larger four-year project called COM-CAP, short for “Community Opioid/Overdose Capacity Building,” that aims to support community overdose response planning in Ontario by building capacity. We conducted the situational assessment between November 2019 and February 2020 as the preparatory phase to inform the development of capacity building supports. Ethics approval was received from Public Health Ontario’s Ethics Review Board (2019–055.01). Verbal (key informant) or implied consent (focus groups and surveys) was obtained from all participants, which was an approved procedure by Public Health Ontario’s Ethics Review Board.

### Study design and setting

**Fig. 1:**
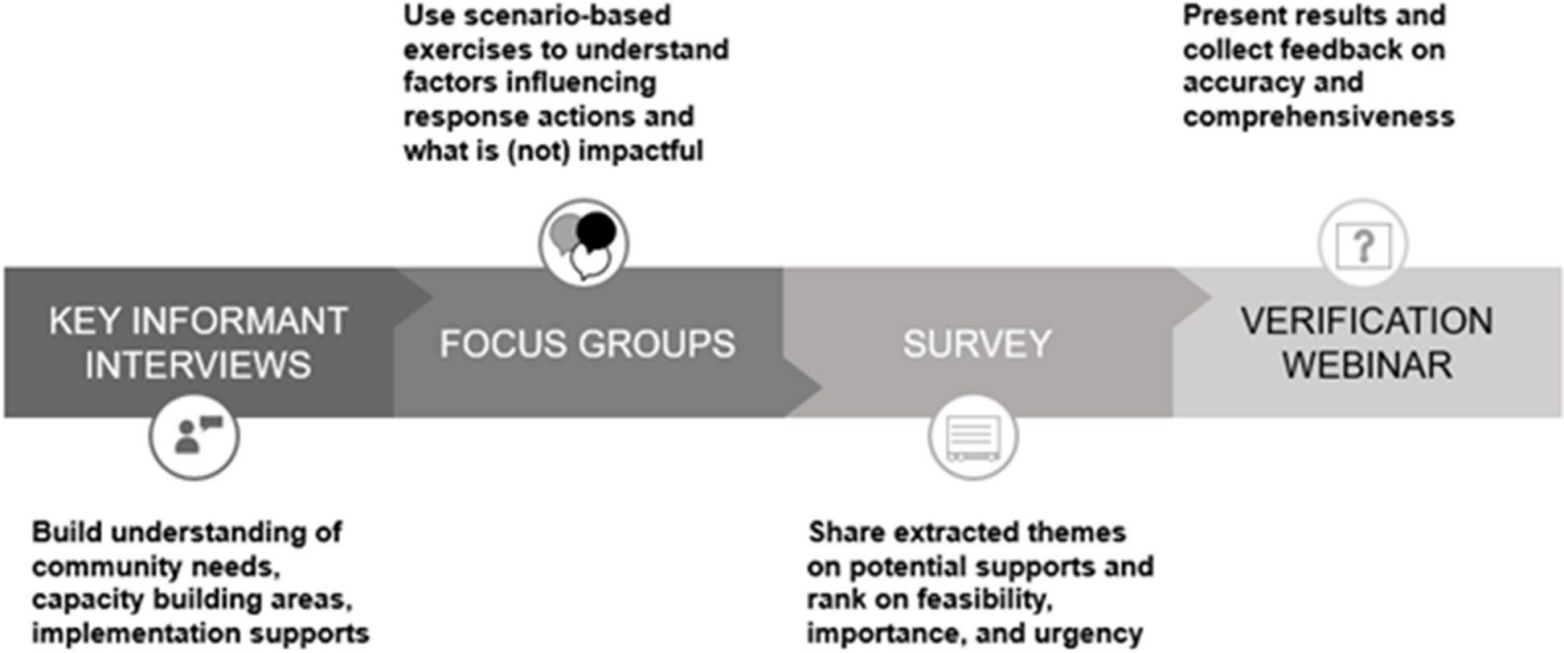
Situational Assessment Activities

Our study applied the six steps of a situational assessment [26] and followed the Standard for Reporting Qualitative Research (SRQR) [27]. Often in health promotion practice [28], situational assessments are used as a comprehensive approach to capture and triangulate data from various data sources to inform action planning [26]. Situational assessments include the following steps: identifying key questions; developing a plan to gather data; gathering the data; communicating findings; and considering how to proceed with planning [26]. In particular, we were interested in understanding the needs of community overdose response plans, the factors that influence the planning and implementation of community overdose response plans, and what actions our project could take to build capacity. We used a mixed methods approach to gather data including interviews and focus groups to elicit in-depth information on the experiences of professionals coordinating community overdose response plans (Fig. 1). The preliminary themes identified in interviews and focus groups were used to generate the items in the survey, permitting for further validation of the themes. Key informant interviews allowed for greater understanding of the various perspectives of stakeholders on the needs for planning and implementation of community overdose response plans. Meanwhile, focus groups captured factors influencing community overdose response plans including what was and was not working well. The methodological approach of our study was similar to other recently published situational assessments conducted at Public Health Ontario [29–31] with the addition of co-design techniques structuring the focus group materials and agenda. Our focus was on professionals coordinating plans to understand this experience, as well as barriers and facilitators, to implementing the community-wide plan, rather than perspectives of all partners and individuals served. While our scope was limited for feasibility, we recognize the importance of diverse perspectives from other partners and particularly community members who use drugs. Our broader project integrates such perspectives through membership on our research team and advisory committee.

Our study was situated in the context of public health practice for substance use in Ontario. In Ontario, the Substance Use Prevention and Harm Reduction Guideline outlines the responsibilities of the 34 local public health units [32]. Within this guideline, and under the Ontario Harm Reduction Program Enhancement, public health units are responsible to lead or support the development of a local overdose response plan [3]. Local drug strategies are involved in addressing substance use-related issues in municipalities using the four pillars of the CDSS and in some cases additional pillars related to integration, housing, or sustaining relationships [33]. Often local drug strategies are hosted at a public health unit, municipal government, or other community-based agency and bring together various community partners such as paramedic services, healthcare, police services, amongst other groups (e.g., local opioid agonist treatment prescribers can contribute to meet needs for access to care). As part of this work, local drug strategies have played a key coordination role on the development of overdose response plans.

### Data collection and participants

#### Key informant interviews

Recruitment of key informants occurred through purposeful sampling, whereby participants were identified through the network of diverse project partners and selected based on their knowledge and experience with supporting, partnering, evaluating, or coordinating community overdose response plans [34]. The sample consisted of 10 key informants, with whom we conducted semi-structured interviews using a guide (See Supplement 1). Key informants were leads of centralised supports at non-for profit organisations, government agencies, or academic institutions; clinician partners; leads of community overdose response plans; and evaluators of community overdose response plans. Interviews lasting 30 to 60 min were conducted by two members of the core project team, digitally recorded, and transcribed verbatim by an external transcriber.

### Focus groups

Using Adobe Connect, we conducted three 2-h focus groups with a total of 25 public health practitioners, drug strategy coordinators, and other leads of community overdose response plans in Ontario (*n* = 9; *n* = 10; *n*= 6 respectively). We invited key contacts at public health units and municipal drug strategies in Ontario working on community overdose response plans. The make-up of each focus group discussion had mixed representation. Each group was facilitated by experienced researchers at the Ontario College of Arts and Design University using a co-design approach that emphasizes the context and experience of key stakeholders as central to a change or innovation process [35]. We asked the participants to describe the responsibilities, influences, and resources available in their role as coordinators of plans, as well as what was working well and not working in different stages of plans (i.e., development, implementation, evaluation). Further, we used scenarios to illustrate situations that participants could see themselves working in to prompt further questions and dialogue. The questions and scenarios can be found in Supplement 2.

### Survey

We developed an online survey using Public Health Ontario’s survey platform. The survey included four main sections, with items related to: 1) potential areas of support; 2) strategies to support implementation; 3) measurements used to evaluate community overdose response plans; and 4) considerations for the selection of community partners for implementing capacity building supports. Each section consisted of closed-end questions that asked to rank different items in terms of feasibility, importance, and urgency, followed by open text to describe the rationale and provide further recommendations (See Supplement 3). Preliminary themes on needs and supports identified from interviews and focus group data informed the items of the survey. Specifically, the section on potential areas of support included nine pre-determined categories reflecting some of the priorities within each of the broader qualitative themes.

The survey was pilot tested with two staff at PHO to ensure clarity of questions and revised before distribution. Invitations to participate in the survey were circulated via email by members of our project team to a list of relevant contacts at public health units (*n* = 38) and municipal drug strategies in Ontario (*n* = 62) involved in community overdose response plans. Participants were asked to collaborate with close partners (e.g., public health units, municipal drug strategy coordinators) to submit one survey per community overdose response plan. The purpose was to reduce duplication and encourage a holistic lens to sharing coordinated efforts. The survey took approximately 30 min to complete. Consent was implied. By clicking on the survey link, participants acknowledged they understood the conditions of participation in the survey and had an opportunity to have questions addressed by project staff.

### Verification webinar

Once we completed the preliminary analysis, we hosted a webinar and invited all municipal drug strategy coordinators and relevant public health staff from Ontario to review our analysis. Coordinators and staff who did not participate in the data collection activities were invited to ensure that the perspectives of those who were unable to participate were captured. We hosted the webinar using Adobe Connect and presented on the themes and subthemes. For each theme, we used the polling feature to ask participants: “how closely does this list reflect needs within the following theme?” To gather further feedback, we used chat boxes whereby participants were able to provide text answers to specific questions including: 1) why do you agree or not agree with the lists/summary?; 2) is there anything you think should be changed?; 3) did we miss anything important and/or critical?; and 4) is there anything you want to add to help us complete our analyses and interpretations?.

### Analysis

#### Researcher characteristics and reflexivity

The study team represents a range of expertise including public health, health design, evaluation, and health and mental health. Three study team members (PL, CBA, TK) were most closely involved in data collection and analysis. Our roles as employees of a provincial public health agency and participants’ potential interest in applying for the project’s community partner funding could have impacted their openness and frankness during key informant interviews and focus groups. To mitigate this potential source of bias, the focus groups were facilitated by a research team member (KS) with less interaction with participants, and input was sought from all study team members on the interpretation and contextualisation of preliminary results.

### Key informant interviews and focus groups

Transcripts from interviews and focus group discussions were reviewed for accuracy and de-identified by a project team member (e.g., all names and other identifying information was removed), and uploaded into the qualitative data analysis software, NVivo 10. Each core project team member (PL, TK, CBA) independently coded a subset of three transcripts and applied thematic analysis for each question with the goal of establishing patterns within the data [36]. This analysis approach was adopted for its inductive process, whereby it does not rely on an existing framework to interpret data [37]. A codebook was then developed through discussion of the differences and additions in codes. Following this, one coder applied this codebook to the remaining transcripts, and another coder independently coded 20% for establishing reliability and consistency. Codes were then grouped together into broader themes and sub-themes, and key quotes expressing most responses were identified.

### Survey

Survey data was imported into Microsoft Excel for analysis. The response rate was not calculated since we do not have information on the number of unique individuals who viewed or started the survey. The intention of the survey was to have it be completed by a community overdose response plan rather than individuals. Descriptive statistics were used to summarise the data collected. For ranking questions, we applied a weighted value and calculated the sum to determine how each item was ranked. Open-ended responses were grouped into common themes. We mapped survey results on needs and related supports to the qualitative themes to understand the commonalities and differences that emerged from the key informant and focus group transcripts.

### Verification webinar

We used descriptive statistics to summarise the data generated through polls on Adobe Connect. Text from chat pods was reviewed and grouped based on similar themes.

### Rigor

We used four techniques to enhance the rigor of our qualitative methods [38]. First, we used multiple methods of data collection to facilitate a more comprehensive understanding of our research question. Second, we adopted a purposive sampling technique to identify appropriate participants providing rich descriptions of community needs and priority supports for community overdose response plans. Third, we used multiple coders to cross check the coding and interpretation of the data by independent project team members. Finally, preliminary themes were presented back to the broader project team and an advisory committee to obtain feedback on the interpretation. As well, these themes were shared with participants in a verification webinar. During the webinar, participants were asked about their reflections of the themes and if anything was missed or misinterpreted. For our quantitative methods, we pilot tested the survey with staff outside of the core project to ensure clarity and that each survey item was distinct. Two core study team members were also involved in applying a weighted value to each ranked item to ensure consistency in the final list.

## Results

In total, 66 participants took part in key informant interviews (*n* = 10), focus groups (*n* = 25), or surveys (*n* = 31). There were two key informant interviews with local coordination roles with potential for overlap in focus group or survey participation. While there was no overlap of participation in key informant interviews and focus groups, local coordinators who participated in key informant interviews or focus groups may have taken part in responding to the survey as the intended purpose was to have one survey completed per community overdose response plan. Overlapping focus group and survey participants are likely given the representation of similar roles in recruitment. Further, 13 people participated in both the focus group and the verification webinar.

We conducted 10 key informant interviews with representatives involved in centralised support for community overdose response or harm reduction (provincial-level in Canada; state-level in the US) (*n* = 5), evaluators of community coalitions/drug strategies (*n* = 2), coordinators of community overdose response plans (*n* = 2), and partner organisations on plans (*n* = 1). Of the 10 key informants, four were from the United States. Focus groups (*n* = 25) and survey respondents (*n* = 31) were coordinators of community overdose response plans in Ontario including public health unit or municipal drug strategy representatives. Twenty-five participants took part in the verification webinar, however, several participants indicated that others were with them on the call. Table 1 provides details of participants’ roles in community overdose response plans We did not collect socio-demographic information during interviews, focus groups, or surveys.

**Table 1:** Participant Roles in Community Overdose Response Plans

Role in Community Overdose Response Plans	No. of participants (*n* = 66)
Ontario-based coordinators (e.g., public health professionals, municipal drug strategy coordinators)	58
Provincial/state-level representatives supporting community overdose response plans	5
Evaluation support	2
Representatives from partner organisations	1

Below, we present results from the interview and focus groups, survey, and verification webinar. First, we describe how the need to address substance use-related stigma (e.g., social, structural) and equity issues as highlighted in both the qualitative and quantitative data. These results are foundational to understanding the themes identified in the qualitative results. For the interview and focus group results, we describe the current implementation practices of community overdose plans and priority areas for supports for capacity building. Four overarching themes for needs and supports emerged related to community overdose response planning: 1) data and information; 2) evidence and practice; 3) implementation/ operational factors; and 4) partnerships, collaboration, and engagement. We discuss the needs identified within each theme. Next, we describe the related capacity building supports for community overdose response planning and implementation across the four themes.

### Stigma and equity

Data from interviews, focus groups, and surveys indicate that stigma and equity are critical for the consideration of all needs and supports related to community overdose response plans. Addressing stigma and equity was described both in the planning process as well as specific strategies and actions included in community overdose response plans. For example, in terms of stigma, participants spoke to the need for specific strategies addressing structural stigma within the healthcare and social service system as well as social stigma towards people who use drugs that influences community support for harm reduction strategies. Meanwhile, within each theme, issues related to equity often centered on the equitable engagement of communities—people with living and lived expertise of substance use and Indigenous and racialised communities—throughout planning process and implementation of community overdose response plans to make sure services better met their needs. Specific gaps were also identified in knowledge and understanding of tailored services, and equity-oriented overdose response actions. For example, some non-Indigenous participants mentioned that more needs to be done to address the colonial lens through which planning and implementation of community overdose response plans take place. Equity-oriented actions aim to address the effects of structural inequities [39–41] on people who use drugs. This includes addressing the effects of intersecting factors that exacerbate racism, discrimination, and stigma experienced by people who use drugs, their access and experiences of services, and the availability of services that meet their social and cultural needs. Culturally safe and trauma-informed approaches are thus aspects of an equity-oriented community overdose response plan [40, 41].

### Interview and focus groups results

#### Current implementation activities

The current implementation and structure of community overdose response plans varied, and reflect activities aligned with common implementation frameworks and guides [42–44]. Most participants mentioned a dedicated coordination role that was key to ensuring consistent work and messaging on plan activities. This role consisted of tasks related to project management, facilitation, and relationship-building with a variety of partners. Other participants spoke of steering committees, advisory boards, or the use of specific leads, workgroups, or sub-committees to organize different plan activities. These organized structures were seen by many to ensure coordination, collaboration, priority-setting, and accountability with the partners. Generally, it was noted that public health infrastructures and personnel were involved as leads or in supportive roles for the ongoing operation of plans.

### Priority needs and related supports

Table 2 provides a summary of the four themes for needs and supports: 1) data and information; 2) evidence and practice; 3) implementation/ operational factors; and 4) partnerships, collaboration, and engagement. We provide an overall description of each theme, priority areas, and exemplar quotes from participants to illustrate interpretations.

**Table 2:** Themes (by alphabetical order), Priority Needs and Related Supports, and Exemplar Quotes

**THEME**	**DATA AND INFORMATION**
Description	Access, use, and communicate data and information. We use the term “data” to mean the collection and presentation of numbers and unit values. By information, we mean the analysis and presentation of data, including evaluation
Priority needs	· Collecting and sharing data · Accessing different sources of local data · Analysing and interpreting data · Using data and information to make decisions and measure the success of plans · Communicating data and information (e.g., alerts, emerging trends)
Related supports	· Provincial coordination and technical assistance in data collection, analysis/interpretation, surveillance, approaches to early warning systems, and communicating data and information
Exemplar quotes	*“So we want to track things, we want to show that what we're doing is making a difference and have our indicators but when we ask our partners for data it's like it's not a priority to them and they don't know where to get it.” (KI6)* *“We still don’t have access to local emergency medical services (EMS) data, even though we’ve been working on a data sharing agreement with them for about a year and a half now.” (FG1)* *“So, of course, the Ministry and ODPRN and PHO have increased sort of data availability, opioid-related data access and different types of data that have supported us in identifying priority populations or areas for action. However, sometimes it's difficult to really prioritize, especially for a community like us that experiences quite a high burden in many areas of morbidity and mortality for opioids. So prioritizing, you know, which information requires the most urgent action and then bringing it into the community coalition. How to kind of show the right community partners and get their support in actually addressing them, who needs to be engaged in certain priorities.” (FG1)*
**THEME**	**EVIDENCE AND PRACTICE**
Description	Access, use, and communicate evidence and best practice. We use the term evidence to refer to information that is gathered for research purposes, contextualized, and used to support a practice, intervention, etc [45].
Priority needs	· Developing knowledge of topics related to overdose response · Accessing research evidence and best practice · Using evidence to inform decision-making and practice · Communicating evidence
Related supports	· Offering training supports · Facilitating access to evidence, common frameworks, e-learning modules, and other tools through a centralised repository · Facilitating consultations and connections to individuals and groups involved in research and practice
Exemplar quotes	*“..So much is being pumped out [laughs] as far as, you know, what publications, lessons learned, you know, all of the above. What we normally find out is it does not always trickle down to the community where it needs to.” (KI2)* *“…which interventions were most effective and which ones might be considered favorable by funding organisations; so yeah we ended up on the internet looking at different things and trying to compare evidence but it was really hard to rank different interventions in terms of their evidence base. So I think this would really help with the evidence base” (KI3)* *“That information is kind of more easily accessible and available rather than having to do it—you know each Public Health Unit or each Drug Strategy Coordinator – on their own. Definitely there’s benefits to ensuring that there’s – you know there’s somewhere we can turn to – like a trusted source for quality information on effective interventions.” (FG1)*
**THEME**	**IMPLEMENTATION/OPERATIONAL FACTORS**
Description	Address the factors that affect the implementation of plans
Priority needs	· Planning strategically · Using consistent approaches to guide development, implementation, and evaluation of plans · Adapting to changing community context · Addressing barriers
Related supports	· Facilitating access to standardised tools, templates, and guides · Facilitating consultation, mentorship, and peer support to other community overdose response plans
Exemplar quotes	*“…people have tended to jump to oh, we’ve been doing this and it’s working so we’re getting money and we’re going to keep doing this, you know, without taking a step back and looking at the data and looking at where we need to build capacity before they … you know, so I think the strategic planning process is really important.”(KI7)* *“…a lot of the organizations are overtaxed to it’s very difficult if you’re trying to bring a group of people together to have them have the time and resources available for this work, I know people are asked to sit on committees all over the place.”(KI3)* *“We’re finding since the development of plan and the implementation of a couple of related working groups is that there’s been a pretty significant shift in focus for the drug strategies based on some other also very pressing issues substance use-related largely around crystal meth.” (FG3)*
**THEME**	**PARTNERSHIPS, COLLABORATION, AND ENGAGEMENT**
Description	Engage, work together, and build trusting partnerships with diverse groups
Priority needs	· Partnerships → identifying partners, building partnerships, navigating, and maintaining partnerships · Collaboration → doing collective and coordinated work · Engagement → ongoing engagement with new sectors, meaningful engagement of people who use drugs, Indigenous communities, and other communities experiencing oppression
Related supports	· Facilitating access to tools for partnership planning and community engagement and resources for developing common visions, goals, and language with community partners · Providing opportunities to connect with diverse groups through online platforms (e.g., online meetings, workshops)
Exemplar quotes	*“I think sometimes as the longevity, whether the drug strategy or the coordinator for the local action plan has been around for some time and it's the ability to maintain relationships. And on the flip side it's once you actually get inroads with some of your community partners and have developed strong relationships, one of the challenges is if they leave” (FG2)* *“These are groups that historically don’t work with each other. And I think there was a lot of misunderstanding as to what the specific roles of each of the individuals were and really what programming was going on in the public safety world and the public health world and how that can be overlapped.” (KI4)* *“I think the biggest need is to try and make sure that decisions that are ultimately affecting people who use drugs and not being made without their voice at the table or in the decision-making process.” (KI5)*

Table 2. Themes (by alphabetical order), Priority Needs and Related Supports, and Exemplar Quotes.

### Theme 1: Data and information

One of the needs and supports we identified is related to local data and information. Of particular concern, was the routine collection and sharing of local data with partners to understand the scope of the problem, needs, and gaps in their community. For example, participants described barriers to the development of data sharing agreements across sectors to gain comprehensive understanding of overdose to inform program development and support evaluation activities with limited resources. Similarly, understanding what local sources of data were available and how to access them was another identified need that posed challenges to understanding overdoses and the impacts of interventions aimed at preventing and addressing overdoses.

Additionally, participants discussed a lack of having appropriate skills and resources to analyse, interpret, and apply data. These areas were viewed as foundational components of strategic planning, early warning, surveillance, and assessing progress and outcomes of plans. A few participants reported on the lack of standardised data indicators and approach to the prioritisation of diverse data that made decision-making for urgent action difficult. Once data was analysed and understood, participants identified the need to better communicate the relevant information to diverse stakeholder using effective, appropriate, and accessible techniques. Discussions on equity and stigma within data and information focused on the need to include and centre the knowledge and experiences of community members and community-based agencies at the forefront of overdose response.

### Theme 2: Evidence and practice

A second theme was the gap in knowledge and use of research evidence to inform strategies included in community overdose response plans. In general, the need to learn about different frameworks and approaches to addressing substance use-related harms, including those that focus on the structural determinants of health (e.g., housing, drug policies, and stigma) was described. An identified barrier to the development of individual knowledge was the limited access to research evidence on effective interventions that reaches the community level where it is needed to inform actions. Further, when access to evidence was available, participants spoke to uncertainty and unfamiliarity with identifying the effectiveness of various solutions within different sectors addressing overdose-related harms.

Next, several participants stated the need to develop understanding of the evidence base related to overdose prevention and response strategies to inform decision-making throughout the development and implementation of community overdose response plans. When evidence was accessible, participants described challenges in understanding which overdose prevention and response strategies have worked well and in which contexts and populations to adapt and tailor strategies (e.g., urban vs. rural contexts). Further, it was noted that the community overdose response context was constantly shifting, and there was uncertainty with incorporating approaches with newer and less clear evidence of effectiveness. Some also pointed out issues related to equity, specifically how knowledge of culturally safe approaches for community overdose was lacking to inform appropriate intervention design and implementation for Indigenous communities. Lastly, participants described the need for support on how to effectively communicate the evidence base of interventions to decision-makers and the public. This included understanding ways to present the evidence into actionable items and use effective channels for different audiences (e.g., websites, social media).

### Theme 3: Implementation and operational factors

Another theme related to specific factors linked to the implementation and operation of community overdose response plans. First, most participants expressed challenges with implementing strategic planning processes related to clarifying scope and work, and organizing and communicating the goals and actions of community overdose response plans (e.g., description of resources, activities, and expected outcomes). Other participants reported on the need to address the coordination of services among different partners within overdose response plans through the use of consistent approaches and standardized provincial tools to guide the strategic planning process. Secondly, for many participants, challenges were identified with the community context in which overdose response plans were situated in, specifically the need to adapt the activities within the plan to the changing community structures, needs, and trends. (e.g., increased methamphetamine use).Many highlighted the gap in integrating an equity lens in programs and services, such as addressing regional inequities and meeting the needs of Indigenous and racialized communities. Participants also emphasized the need to communicate and share plans with stakeholders as a means of increasing trust and engagement. Dependent on the sociopolitical community context, needs around strategizing to communicate plans with stakeholders were also articulated. Finally, many participants identified the need for supports to address implementation barriers, including limited resources (e.g., financial, human) and time, substance use stigma and community buy-in, and infrastructure.

### Theme 4: Partnerships, collaboration, and engagement

The last theme pertained to the partnerships, collaboration, and engagement of community within overdose response plans. According to participants, sectors responding to overdose and related harms often operated in silos and had limited pre-existing structures and experience working together. This presented difficulties navigating local overdose-related activities and identifying and establishing trusting partnerships with key sectors (e.g., healthcare, pharmacy, law enforcement). Some described the competition for resources, lack of communication, unwillingness, and the different disciplines of sectors as contributing factors to the barriers to building partnerships. Further to this were concerns around how to work collaboratively with partners outside of a mandate. Several participants expressed that effort was needed to develop an awareness and understanding of the terminology used by each sector to establish a shared framing, build consensus, prioritise, and coordinate intervention strategies with multiple partners.

Participants also commented on the need for greater equitable engagement and outreach to diverse stakeholders for representation in community overdose response plans. In particular, the meaningful engagement of people with living and lived expertise of substance use was seen as lacking. Despite favorable views of engagement with people with living and lived expertise of substance use, capacity, resources, and stigma were commonly highlighted as barriers in doing so. Others mentioned the need to build capacity to engage and partner with Indigenous and racialised communities and organisations to develop specific recommendations that appropriately meet their needs.

### Supports to address needs across themes

Often, suggested supports were relevant and applicable to addressing the four different themes. For example, a common support emphasized by participants was the access to standardized tools and templates, planning and implementation frameworks (e.g., guide the design, delivery, and evaluation of substance use practices), and educational and other resources (e.g., for developing a common vision, goal, and language with community partners) through centralised repository for coordinators and partners of community overdose response plans. In particular, participants suggested standardised tools and templates for: partnership planning and community engagement; decision-making and prioritisation; action planning and environmental scans; and monitoring and reporting plan outcomes.

Other support strategies included consultations, connections, and online platforms (e.g., online meetings, workshops) to facilitate access to and use of evidence as well as collaboration and engagement with diverse groups responding to overdose-related harms. Mentorship and peer support from other community overdose response plans was also suggested to help address barriers and adapt tools and strategies to local contexts. To develop knowledge, training supports and e-learning modules were noted in the areas of harm reduction, trauma-informed approaches, knowledge translation, evaluation, stigma, cultural safety, community engagement and development, and evidence-informed practices. Lastly, provincial coordination and technical assistance were suggested for data collection, analysis, surveillance, early warning systems, and identifying and implementing strategies to communicate local data and information (e.g., alerts, emerging trends).

### Survey results

A total of 31 respondents completed the survey, with 26 of 34 public health units in Ontario represented. Table 3 summarises the characteristics of survey respondents.

**Table 3:** Characteristics of Survey Respondents

Characteristic	Responses *n* = 31(%)
Organization	
Public Health Unit	16 (51.6)
Combined Public Health Unit/Municipal Drug Strategy	10 (32.3)
Municipal Drug Strategy	3 (9.7)
Other	2 (6.5)
Region of Ontario	
South Central	9 (29.0)
South West	9 (29.0)
North	7 (22.6)
South East	6 (19.4)

When asked to rank the top five pre-determined areas related to overdose response plans for capacity building supports, the findings were ordered accordingly: evidence use and application, program implementation, community engagement, knowledge and skill development, and partnership and collaboration, (See Table 4). Respondents were also asked to describe any other areas for support to their overdose response plans. Open-ended responses to this question were grouped into five categories reflecting similar themes: 1) provincial coordination and alignment with sectors to address overdose and determinants of health issues; 2) data and surveillance including early warning, alert communication, and real-time provincial data collection systems involving all community partners; 3) professional development opportunities for staff involved in local overdose-related work; 4) equity considerations to plan for and meet the access needs of diverse communities including Indigenous peoples; and 5) policy and funding supports for overall implementation of community overdose response plans as well as to scale up key harm reduction and treatment services.

**Table 4:** Weighted Ranking of Top Five Areas for Capacity Building Supports

Qualitative Themes	Survey Support Area	Unweighted Rank *(simple count rating)*		Weighted Rank *(weighted point rating)*	
		Count	Ranking	Sum	Ranking
Evidence and practice	Evidence use and application	27	1	84	1
Partnership, collaboration, and engagement	Community engagement	21	2	61	3
Implementation and operational factors	Public awareness and education	19	3	-	-
Implementation and operational factors	Program implementation	18	4	64	2
Evidence and practice	Knowledge and skill development	16	5	49	4
Data and information	Evaluation capacity	15	-	-	-
Partnership, collaboration, and engagement	Partnership and collaboration	15	-	47	5
Data and information	Data collection	14	-	-	-
Data and information	Data analysis and interpretation	10	-	-	-

The areas of capacity building supports were informed by the preliminary themes from interview and focus group data. To calculate the weighted rank, the following rank value was assigned: 1 = 5, 2 = 4, 3 = 3, 4 = 2, 5 = 1

Table 4. Weighted Ranking of Top Five Areas for Capacity Building Supports.

### Verification webinar

When asked how closely each theme reflects the needs of municipal drug strategy coordinators and relevant public health staff, there was strong agreement with the four main themes and subthemes. Table 5 presents how closely our analysis reflects the needs of participants. The *partnership, engagement, and collaboration* had the lowest agreement, with 50% of participants reported that there were a few items missing. One of the main items identified in the verification phase that was not elucidated previously was the need to improve accountability of partnerships with implementing the actions. In terms of other themes, data quality and fidelity, technology options to administer overdose alerts, and evidence related to the sustainability of community overdose response plans were suggested as other considerations to inform our interpretation.

**Table 5:** Verification of Preliminary Analysis

Theme	Responses: How closely does this list reflect needs within the following theme?	n (%)*
Data and information	*Very well*	13 (81%)
	*You have most things but a few are missing*	3 (19%)
	*I’d agree with about half of this list*	0
	*I don’t think the slide is reflective at all*	0
Evidence and practice	*Very well*	11 (92%)
	*You have most things but a few are missing*	1 (8%)
	*I’d agree with about half of this list*	0
	*I don’t think the slide is reflective at all*	0
Implementation/operational factors	*Very well*	11 (85%)
	*You have most things but a few are missing*	2 (15%)
	*I’d agree with about half of this list*	0
	*I don’t think the slide is reflective at all*	0
Partnership, collaboration, and engagement	*Very well*	9 (50%)
	*You have most things but a few are missing*	9 (50%)
	*I’d agree with about half of this list*	0
	*I don’t think the slide is reflective at all*	0

^*^Note: Not all participants are represented as some did not vote in the verification polls. The total number of participants that participated in each poll question varies

Table 5. Verification of Preliminary Analysis.

## Discussion

To our knowledge, this is the first situational assessment of the needs and priorities of communities developing and implementing overdose response plans. The current practice and structure of community overdose response plans vary in Ontario, and in other communities in Canada and the US. For example, local plans in Canada often refer to the Four Pillar Drug Strategy approach (i.e., prevention, treatment, harm reduction, enforcement) [2], many in the US align with the Project Lazarus hub and spoke model, with 10 areas of focus including coalition action and prescription medication diversion control [24], We found four common themes of needs and related supports were identified across plans in Ontario. These themes related to evidence and practice; data and information; implementation/operational factors; and partnerships, engagement, and collaboration. Needs for supports related to access, use, and communication of data, information, evidence and best practices on overdose and response strategies were also expressed. Current strategic planning processes, service coordination, standardization, and broader contextual factors were reportedly affecting the implementation/operation of community overdose response plans. Participants had challenges engaging, building trusting partnerships, and working collaboratively with diverse sectors and organisations involved in community overdose response plans. A consistent need to address stigma and equity issues in community overdose response plan was illustrated throughout all four themes.

Our findings highlight similarities with and map well onto common components of capacity building frameworks [9, 46] such as training, tools, and technical assistance [47]. The four themes that were determined in our study also aligned with those from situational assessments on other public health practices in Ontario, specifically needs related to data, evidence, and collaboration [29–31]. Such consistencies across other areas underscore the need for broader capacity building efforts to support public health actions in Ontario [29–31].

Furthermore, our findings are consistent with barriers to community overdose action [25] and strategies used by centralised structures supporting them such as provision of standardised tools, training, strategic planning support, and assistance with data and evaluation [20, 21, 48]. While the need to address and reduce structural and social stigma related to substance use [49] was presented in some centralised structures, a key theme that was integrated throughout our findings and lacking in others was the need to address equity related to planning and implementing community overdose response plans. It is known that the impacts of substance use-related stigma are exacerbated when intersecting with multiple forms of discrimination including systemic racism, sexism, and other forms of oppression connected to marginalization [50]. Our findings reinforce the growing evidence and support the need to address structural inequities in public health planning and practice in the context of substance use-related harms [41, 50–52].

### Implications for practice

Within the context of the continually evolving overdose crisis, limited resources, and constantly shifting challenges, it is critical to understand where to focus supports for the development and implementation of community overdose responses in order to maximise impact on overdose-related harms. Our study asked potential recipients of supports what needs to be addressed, how to best address these, and what should be prioritised (i.e., data and information; implementation/operational factors; and partnerships, engagement, and collaboration; Table 2). In describing the priority needs and related supports, our analysis offers insights into the key components for building capacity of community-based overdose response in Ontario.

While there is variation in the planning and implementation contexts of community overdose response plans, the common themes identified, including the standardisation of data indicators, tools, and frameworks, demonstrate the potential role of centralised supports that can be adapted to local practice contexts. Community overdose response plans further emphasized the need for formalized provincial structures for coordination and knowledge sharing. These results will inform the next phase of our project – gathering detailed requirements for the design of capacity building supports through a multi-stakeholder co-design workshop (e.g., designing shared templates for stakeholder meetings or resource-sharing website). In recognising that community overdose response plans take place within complex organizational, clinical, and community environments, one area for further investigation will be at which level central capacity building supports be provided for community overdose response plans (e.g., coordinator-level). Once key supports have been detailed and developed together with community partners, we will work closely with three community initiatives in Ontario to implement and evaluate the supports. Further research and evaluation will be needed to improve our understanding of how components of our supports have influenced processes at the community-level, and to look for any positive or negative unintended consequences.

### Strengths and limitations

A key strength was the study design and the use of multiple methods and sources. As such, our analysis allowed for a nuanced understanding of the planning and implementation of community overdose response plans, and highlighted similarities and differences in the data generated by each method. We primarily sought the perspective of those involved in coordination of community overdose response plans, which helps make sure the needs of key stakeholders are reflected in the development of supports for plan development and implementation. The involvement of other stakeholders supporting or partnering on community overdose responses plans in various regions across Canada and the U.S. allowed for a wider range of perspectives. The use of verification techniques with data collection and analysis also strengthened the rigor of our study and findings.

The limitations of our study should also be noted. First, our study reflects the Ontario context. Community overdose response plans elsewhere may operate differently, and our results do not reflect the range of needs and areas for capacity building supports in other jurisdictions. Other limitations were the small number of interviews and the depth of the focus group discussion due to time constraints from the project timelines. Our study focused primarily on the needs of professionals coordinating community overdose response plans including drug strategy coordinators. As such, the experiences of people with living and lived expertise of substance use were not captured and may not align with the priorities of other stakeholders. However, people with living and lived expertise of substance use were involved in interpreting the preliminary findings and their involvement will be ongoing with the design of support strategies including the multi-stakeholder workshop.

Since these data were collected, the nature of the overdose crisis and the challenges experienced by those responding may have evolved from the data presented here. Although the number of overdose deaths was increasing preceding the coronavirus pandemic (COVID-19), recent data highlights worsening rates since the start of the pandemic [53]. The increasing toxicity of the unregulated drug supply [53], service disruptions to harm reduction programs from public health measures, and the deployment of public health workforce to the COVID-19 response have impacted the landscape for community overdose response plans including the emergence of new stakeholders and strategies. As overdose deaths have escalated in this context, there has been increased focus and action on approaches such as decriminalization of drug possession for personal use and provision of a predictable supply of drugs as an alternative to the toxic drug market. Accordingly, there is a need to continue building capacity for sharing local information and experiences on innovative approaches.

## Conclusion

Community overdose response plans have emerged to prevent and address overdose-related harms across communities in Ontario; however, there are considerable practice needs that may influence implementation. By understanding the needs and related support across community overdose response plans in Ontario, we can identify essential and responsive strategies to build local capacity to address overdose-related harms. Centralised structures can support common needs experienced across community overdose response plans and help facilitate the adoption of evidence-based practices to improve the provision of care and ultimately impact overdose-related morbidity and mortality. Ongoing evaluation of our project and other centralised structures supporting overdose-related initiatives will highlight the effect of supports on the efforts of community overdose response plans.

## Supplementary Information

**Additional file 1. **Supplement 1 

**Additional file 2.** Supplement 2

**Additional file 3. **Supplement 3

## Data Availability

The interview and focus group transcripts that were analysed for our study will not be made publically available. This is to ensure the privacy and confidentiality of our study participants is protected. Similarly, the data generated and analysed from the survey has data that could be potentially identifiable.
