# Supplementary material for: Supporting community overdose response planning in Ontario, Canada: Findings from a situational assessment
Source: BMC Public Health. 2022 Jul 19;22:1390. doi: 10.1186/s12889-022-13762-0 (PMC9296108; doi:10.1186/s12889-022-13762-0)
Supplement: Supplementary file 1 — Additional file 1. Supplement 1 [file 12889_2022_13762_MOESM1_ESM.docx]

**Supplement 1. Key Informant Interview Guide**

1. What is your [organization’s] current role/involvement in addressing opioid/overdose-related harms in the community?
   1. **Probe:** How do you/your organization go about developing and implementing opioid/overdose-related work?
2. Reflecting on your experience, what are the greatest needs of community coalitions developing opioid/overdose related plans?
3. What are the greatest priorities of community coalitions developing opioid/overdose-related plans?
   1. **Probe:** What do you think are current opportunities to strengthen community opioid/overdose-related work in Ontario?
   2. **Probe:** What worries you about your opioid/overdose-related work [in your community]?
4. What types of technical and capacity-building supports would assist/be helpful in developing and implementing community opioid/overdose-related plans?
   1. **Probe:** What key tools or supports are community coalitions currently using for opioid/overdose-related work?
   2. **Probe:** What gaps exist in the tools and supports available to you for reference and use?
5. Among the suggestions, what would you highlight as the top 2-3 priorities for us to focus on?
   1. **Probe:** How do you think this support would make a difference?
   2. **Probe:** Are there any current barriers to accessing existing available supports?
6. What would you suggest we consider when we select our pilot sites?
   1. **Probe:** What would you suggest we consider in terms of capacity? What about readiness?
   2. **Probe:** What about internal or external factors that could influence a site’s ability to adapt/implement the model?
   3. **Probe:** Are there any critical factors/characteristics to look for?
7. What internal and external factors do you think would influence implementation?
8. What types of strategies would help support/facilitate implementation of the model components?
   1. **Probe:** What challenges do you foresee in the implementation of the model for community opioid/overdose-related plans?
9. What are important process indicators to track progress?
   1. Examples of process indicators could include the number of resources distributed or number of activities and people participating in activities, programs, and services.
10. What are important outcome indicators to measure?
    1. Examples of outcomes could include the number of deaths related to opioids or number of emergency department visits.
11. [Show dashboard mock-up] – How might this resource support your work?
    1. **Probe:** How would you use this resource in practice?
    2. **Probe:** In your opinion, what is missing from this resource? How might this resource not meet your needs?
12. Who else do you recommend we speak to regarding supports for community opioid/overdose-related plans in Ontario?
13. Do you have any other suggestions or feedback that has not yet been covered in our discussion?
