# Supplementary material for: Supporting community overdose response planning in Ontario, Canada: Findings from a situational assessment
Source: BMC Public Health. 2022 Jul 19;22:1390. doi: 10.1186/s12889-022-13762-0 (PMC9296108; doi:10.1186/s12889-022-13762-0)
Supplement: Supplementary file 2 — Additional file 2. Supplement 2 [file 12889_2022_13762_MOESM2_ESM.docx]

**Supplement 2. Focus group questions and scenarios**

1. What are the most important responsibilities of drug strategy coordinator in opioid/overdose-related plans?
2. What are your main resources (skills, tools, funding sources…) to support the opioid/overdose-related plans?
3. How do you influence the implementation of the opioid/overdose-related plans among sector partners?

**Scenario 1:** Roy, drug strategy coordinator, municipality within drug strategy, part-time, funding from a foundation, rural area –**Harm Reduction**

**“I have weak support from leadership, including the mayor.”**

Roy works as a drug strategy coordinator in supporting the implementation of **harm reduction services** (e.g., supervised consumption sites, overdose prevention sites) in a **conservative, rural community** that spreads over a large geographic region.

In this scenario (from your experience):

1-What is working well?

2-What is not working? (Gaps and pain points)

3-What are your workarounds?

4-What resources do you have? What do you need to have?

5-What would you expect in terms of support?

**Scenario 2:** Jasmine, the local opioid action plan coordinator, public health unit, full-time, funding from the ministry of health and Long-term care,major urban area - **Treatment**

**“The barriers to accessing healthcare are frustrating, and there seems to be a disconnect with other services.”**

Jasmine is focused on improving coordination and promoting evidence-based guidelines, health system quality standards, educational and training opportunities, and knowledge translation tools to achieve improved care for mental health and substance use.

In this scenario (from your experience):

1-What is working well?

2-What is not working? (Gaps and pain points)

3-What are your workarounds?

4-What resources do you have? What do you need to have?

5-What would you expect in terms of support?

**Scenario 3:** Nita, municipal drug strategy coordinator, public health unit, part-time, permanent with secure funding, urban/rural area - **Prevention**

**“Working with social housing is a challenge, we’ve seen a lot of stigma-related decisions, community support is mixed.”**

Nita is focused on supporting the coordination and implementation of **prevention programs** (e.g. disposing prescription medication, prevention programs for youth and family, **social determinants of health**) in a **mixed rural/urban** geographic area

In this scenario (from your experience):

1-What is working well?

2-What is not working? (Gaps and pain points)

3-What are your workarounds?

4-What resources do you have? What do you need to have?

5-What would you expect in terms of support?

**Plan Development**

1-What is working well?

2-What is not working? (Gaps and pain points)

3-What are your workarounds?

4-What resources do you have? What do you need to have?

5-What would you expect in terms of support?

**Probes:** assessment of the risk and protective factors; enhancing the community readiness; providing a collaborative platform; community structure and diversity; the social and cultural framework

**Plan Implementation**

1-What is working well?

2-What is not working? (Gaps and pain points)

3-What are your workarounds?

4-What resources do you have? What do you need to have?

5-What would you expect in terms of support?

**Probes:** prioritizing and tailoring implementation strategies; collecting data and information; local community access to opioid-related knowledge; local community access to social skills; local access to services

**Community Coalition**

1-What is working well?

2-What is not working? (Gaps and pain points)

3-What are your workarounds?

4-What resources do you have? What do you need to have?

5-What would you expect in terms of support?

**Probes:** community awareness and involvement; collaboration, support, and leadership; diversified funding sources; inclusivity, engagement, and integrity; context sensitive solutions

**Plan Evaluation**

1-What is working well?

2-What is not working? (Gaps and pain points)

3-What are your workarounds?

4-What resources do you have? What do you need to have?

5-What would you expect in terms of support?

**Probes:** monitoring outcome indictors such as deaths, prescriptions…; monitoring process indicators such as policies, guidelines…; coupling policymaking with evaluation plans; surveillance, monitoring, and reporting; access to real-time health data

**Plan Evaluation**

1-Which parameters do you think we should consider for selecting pilot sites?

**Probes:** severity of problem; community awareness and support; leadership support; stage of activities and efforts; obtaining resources

Is there anything else you would like to discuss or share with us?
