# Supplementary material for: Supporting community overdose response planning in Ontario, Canada: Findings from a situational assessment
Source: BMC Public Health. 2022 Jul 19;22:1390. doi: 10.1186/s12889-022-13762-0 (PMC9296108; doi:10.1186/s12889-022-13762-0)
Supplement: Supplementary file 3 — Additional file 3. Supplement 3 [file 12889_2022_13762_MOESM3_ESM.docx]

**Supplement 3. Survey Questions**

**1. Please select if you are responding on behalf of a public health unit or municipal drug strategy:**

Public health unit

Municipal drug strategy

Combined public health unit/drug strategy

Other: ______________________

**2.**  **What area of Ontario do you work in?**

North: Sudbury, North Bay or anywhere north of those cities (including Thunder Bay)

Southwest: Guelph, Brantford or anywhere west of those cities (including Kitchener-Waterloo-Cambridge, London, Windsor)

South Central: GTA, Niagara, Barrie, Muskoka

Southeast: Peterborough or anywhere east of there (including Kingston, Ottawa)

**3. The list below describes potential areas of support for community opioid-related plans in Ontario. Please select the top five potential areas of supports from the list below. Rank them in order of importance (most important at the top) based on the following criteria:**

- **Urgency/timeliness** (Is this area of support required in a timely manner for community opioid-related plans in Ontario?)
- **Importance** (Is this area of support needed for community opioid-related plans in Ontario?)
- **Impact** (Would this area of support make a significant difference for community opioid-related plans in Ontario?)

1. Evaluation capacity
2. Evidence use and application
3. Knowledge and skill development
4. Partnership and collaboration
5. Data collection
6. Program implementation
7. Public awareness and education
8. Community engagement
9. Data analysis and interpretation

**4. Can you please briefly describe why you ranked the above areas of support this way?**

**5. Are there any potential areas of supports missing from the list above?**

Yes

No

**If yes, please list any additional items that you feel should be included (if multiple items please number them).If no, please leave the text box blank.**

**6. The list below describes strategies that can be used to support the implementation of community opioid-related plans. Please select the top five implementation strategies from the list below. Rank them in order of importance (most important at the top) based on the following criteria:**

- **Feasibility**(Is there adequate time, resources, and personnel to implement the strategy?)
- **Importance**(Is this strategy essential for implementing community opioid-related plans in Ontario?)

1. Train-the-trainer
2. Use of advisory boards, workgroups or subcommittees
3. Develop resource sharing agreements
4. Local champions
5. Involve people with lived experience including family members
6. Capture and share local knowledge
7. Conduct local consensus discussions
8. Ongoing training
9. Involve leadership/executive boards
10. Ongoing consultation
11. Tailor strategies
12. Recruit and train coordinators

**7. Can you please briefly describe why you ranked the above strategies this way?**

**8. Are there any potential implementation strategies missing from the list above?**

Yes

No

**If yes, please list any additional items that you feel should be included (if multiple items please number them).If no, please leave the text box blank.**

**9. The list below describes some of the measurements used to evaluate community opioid-related plans. Please select the top five measurements for a comprehensive evaluation. Rank them in order of importance (most important at the top).**

1. Number of opioid-related deaths
2. Number of opioid-related hospitalization
3. Number of opioid-related emergency department visits
4. Number of individuals on opioid agonist treatment
5. Awareness and support of community opioid plan
6. Number of reported youth uptake of substance use
7. Naloxone administration
8. Number of EMS opioid-related calls
9. Number of opioid-related police calls
10. Local crime rates
11. Attitudes towards substance use
12. Awareness, access, and uptake of harm reduction services

**10. Are there any considerations missing from the list above?**

Yes

No

**If yes, please list any additional items that you feel should be included (if multiple items please number them). If no, please leave the text box blank.**

**11. The list below describes considerations for selection of pilot sites in Ontario. Please select the top three considerations for site selection from the list below. Rank them in order of importance (most important at the top).**

- **Community climate:**The prevailing attitude of the community towards addressing opioid-related harms is supportive.
- **Community knowledge about the issue:** Community members are knowledgeable about the causes of increasing opioid-related harms and the impacts on community.
- **Leadership:** Appointed leaders and influential community members are supportive of the issue.
- **Community efforts:** There are community opioid-related efforts, programs, and policies in place.
- **Community knowledge of the efforts:** Community members are knowledgeable about local efforts to reduce opioid-related harms.
- **Resources related to the issues:** There are people, funds, and other resources available to support community opioid-related efforts.
- **Community impact:** Rates of opioid-related morbidity and mortality.

**12. Can you please briefly describe why you ranked the above considerations this way?**

**13.  Are there any considerations missing from the list above?**

Yes

No

**If yes, please list any additional items that you feel should be included (if multiple items please number them). If no, please leave the text box blank.**

**Thank you for completing the survey!**
